# (*E*)-1-(Furan-2-yl)-3-(2,4,5-trimeth­oxy­phen­yl)prop-2-en-1-one

**DOI:** 10.1107/S1600536812000037

**Published:** 2012-01-11

**Authors:** Thitipone Suwunwong, Suchada Chantrapromma, Chatchanok Karalai, Pitikan Wisitsak, Hoong-Kun Fun

**Affiliations:** a Crystal Materials Research Unit, Department of Chemistry, Faculty of Science, Prince of Songkla University, Hat-Yai, Songkhla 90112, Thailand; b Excellence Center, Mae Fah Luang University, Thasud, Muang, Chaing Rai 57100, Thailand; c X-ray Crystallography Unit, School of Physics, Universiti Sains Malaysia, 11800 USM, Penang, Malaysia

## Abstract

In the title chalcone derivative, C_16_H_16_O_5_, the dihedral angle between the furan and benzene rings is 2.06 (17)°. The two meth­oxy groups at the *ortho* and *para* positions are essentially coplanar with the benzene ring [C—O—C—C angles = −1.0 (5) and 178.5 (3)°], whereas the third one at the *meta* position is slightly twisted [C—O—C—C = 9.6 (5)°]. In the crystal, weak C—H⋯O inter­actions link the mol­ecules into a sheet parallel to (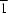
02). An inter­molecular π–π inter­action between the furan and benzene rings is present [centroid–centroid distance = 3.772 (2) Å]. A short C⋯C contact [3.173 (5) Å] is also observed between neighbouring furan rings.

## Related literature

For background to and applications of chalcones, see: Cheng *et al.* (2008 ▶); Jung *et al.* (2008 ▶); Lee *et al.* (2006 ▶); Liu *et al.* (2011 ▶); Nerya *et al.* (2004 ▶); Suwunwong *et al.* (2011 ▶); Tewtrakul *et al.* (2003 ▶). For related structures, see: Fun *et al.* (2010*a* ▶,*b* ▶, 2011 ▶). For the stability of the temperature controller, see: Cosier & Glazer, (1986 ▶). For standard bond-length data, see: Allen *et al.* (1987 ▶).
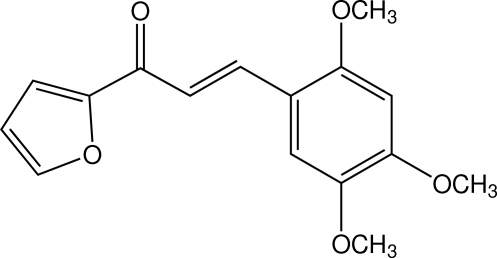


## Experimental

### 

#### Crystal data

C_16_H_16_O_5_
*M* *_r_* = 288.29
Monoclinic, 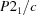

*a* = 8.338 (2) Å
*b* = 8.610 (2) Å
*c* = 18.923 (5) Å
β = 94.467 (4)°
*V* = 1354.4 (6) Å^3^
*Z* = 4
Mo *K*α radiation
μ = 0.11 mm^−1^
*T* = 100 K
0.28 × 0.21 × 0.09 mm

#### Data collection

Bruker APEX DUO CCD area-detector diffractometer
Absorption correction: multi-scan (*SADABS*; Bruker, 2009 ▶) *T*_min_ = 0.971, *T*_max_ = 0.991
8001 measured reflections
2647 independent reflections
1663 reflections with *I* > 2σ(*I*)
*R*_int_ = 0.068

#### Refinement

*R*[*F* ^2^ > 2σ(*F* ^2^)] = 0.064
*wR*(*F* ^2^) = 0.175
*S* = 1.08
2647 reflections
193 parameters
H-atom parameters constrained
Δρ_max_ = 0.33 e Å^−3^
Δρ_min_ = −0.40 e Å^−3^

### 

Data collection: *APEX2* (Bruker, 2009 ▶); cell refinement: *SAINT* (Bruker, 2009 ▶); data reduction: *SAINT*; program(s) used to solve structure: *SHELXTL* (Sheldrick, 2008 ▶); program(s) used to refine structure: *SHELXTL*; molecular graphics: *SHELXTL*; software used to prepare material for publication: *SHELXTL* and *PLATON* (Spek, 2009 ▶).

## Supplementary Material

Crystal structure: contains datablock(s) global, I. DOI: 10.1107/S1600536812000037/is5037sup1.cif

Structure factors: contains datablock(s) I. DOI: 10.1107/S1600536812000037/is5037Isup2.hkl

Supplementary material file. DOI: 10.1107/S1600536812000037/is5037Isup3.cml

Additional supplementary materials:  crystallographic information; 3D view; checkCIF report

## Figures and Tables

**Table 1:** Hydrogen-bond geometry (Å, °)

*D*—H⋯*A*	*D*—H	H⋯*A*	*D*⋯*A*	*D*—H⋯*A*
C14—H14*B*⋯O2^i^	0.96	2.47	3.363 (5)	154
C15—H15*C*⋯O1^ii^	0.96	2.43	3.365 (4)	165

Symmetry codes: (i) 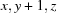; (ii) 
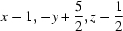
.

**Fig. 1.**
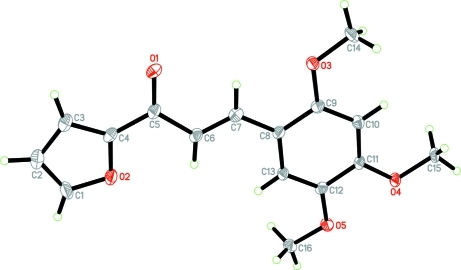
The molecular structure of the title compound, showing 50% probability displacement ellipsoids.

**Fig. 2.**
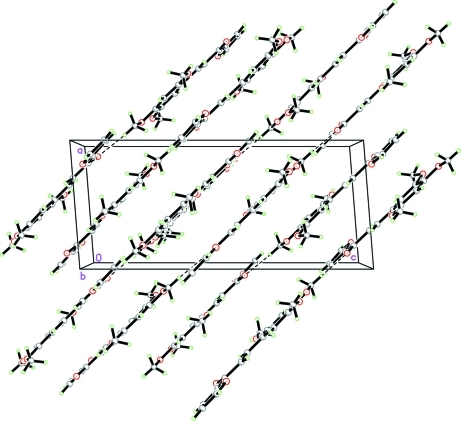
The crystal packing of the title compound viewd along the b axis, showing molecular sheets parallel to the (1 0 2) plane. Hydrogen bonds are shown as dashed lines.

## supplementary crystallographic information

### Comment

Chalcones and heteroaryl chalcones have been reported to possess a wide range of biological activities such as antibacterial (Liu *et al.*, 2011), anti-inflammatory (Lee *et al.*, 2006), anti-oxidant (Cheng *et al.*, 2008), HIV-1 protease inhibitory (Tewtrakul *et al.*, 2003) as well as anti-tyrosinase activities (Nerya *et al.*, 2004), including fluorescent property (Jung *et al.*, 2008; Suwunwong *et al.*, 2011). The title heteroaryl chalcones (I) was synthesized to study for its fluorescence property and tyrosinase inhibitory activity and also to compare its property with the previously published related compounds (Suwunwong *et al.*, 2011). Our experiment shows that (I) exhibits fluorescence property (Suwunwong *et al.*, 2011) and tyrosinase inhibitory activity with the IC_50_ value of 0.143±0.002 mg.ml^-1^. Herein the crystal structure of (I) is reported.

The molecule of (I) in Fig. 1 exists in an *E* configuration with respect to the C6═C7 double bond [1.335 (5) Å]. The molecule is planar with the dihedral angle between the furan and the benzene rings being 2.06 (17)°. The middle prop-2-en-1-one unit (O2/C5–C7) is also planar with the *r.m.s*. 0.0013 (2) and the torsion angle O1–C5–C6–C7 = -0.4 (5)°. The mean plane through this unit makes dihedral angles of 4.1 (2)° and 3.6 (2)° with the furan and the benzene rings, respectively. The two methoxy groups at *ortho* (at atom 9) and *para* (at atom C11) positions of 2,4,5-trimethoxyphenyl unit are essentially co-planar with the attached benzene ring with torsion angles C14–O3–C9–C10 = -1.0 (5)° and C15–O4–C11–C12 = 178.5 (3)°, whereas the third one at *meta* (at atom C12) position is slightly twisted with the torsion angle of C16–O5–C12–C13 = 9.6 (5)°. These angle values also indicated that the methyl group at *para* position points toward the one at *ortho* but point away from the one at *meta* positions due to the sterric effect. The bond distances have normal values (Allen *et al.*, 1987) and are comparable with closely related structures (Fun *et al.*, 2010*a*,*b*, 2011).

In the crystal packing (Fig. 2), weak C14—H14B···O2^i^ and C15—H15C···O1^ii^ interactions (Table 1) link the molecules into sheets parallel to the (1 0 2) plane and these sheets are stacked along the *a* axis by π–π interactions with *Cg*1···*Cg*2^iii^ = 3.772 (2) Å [symmetry code: (iii) 1-x, 2-y, 1-z]; *Cg*1 and *Cg*2 are the centroids of C1–C4/O2 furan and C8–C13 benzene rings, respectively. A C1···C1^iv^[3.173 (5) Å; symmetry code: (iv) 2-x, 1-y, 1-z] short contact is also observed.

### Experimental

The title compound was prepared by the condensation of the solution of 2-furyl methylketone (2 mmol, 0.22 g) in ethanol (15 ml) with the solution of 2,4,5-trimethoxybenzaldehyde (2 mmol, 0.40 g) in ethanol (15 ml) in the presence of 20% NaOH (aq) 5 ml at 278 K for 4 hr. The resulting solid which was obtained was collected by filtration, washed with distilled water and dried in air. Yellow slab-shaped single crystals of the title compound suitable for X-ray structure determination were recrystalized from acetone:ethanol (1:1 *v*/*v*) by the slow evaporation of the solvent at room temperature after several days (m.p. 356–357 K).

### Refinement

All H atoms were positioned geometrically and allowed to ride on their parent atoms, with *d*(C—H) = 0.93 Å for aromatic and CH, and 0.96 Å for CH_3_ atoms. The *U*_iso_(H) values were constrained to be 1.5*U*_eq_ of the carrier atom for methyl H atoms and 1.2*U*_eq_ for the remaining H atoms. A rotating group model was used for the methyl groups. The highest residual electron density peak is located at 0.83 Å from C10 and the deepest hole is located at 0.89 Å from C4.

### Crystal data

**Table:**

C_16_H_16_O_5_	*F*(000) = 608
*M**_r_* = 288.29	*D*_x_ = 1.414 Mg m^−^^3^
Monoclinic, *P*2_1_/*c*	Melting point = 356–357 K
Hall symbol: -P 2ybc	Mo *K*α radiation, λ = 0.71073 Å
*a* = 8.338 (2) Å	Cell parameters from 2647 reflections
*b* = 8.610 (2) Å	θ = 2.2–26.0°
*c* = 18.923 (5) Å	µ = 0.11 mm^−^^1^
β = 94.467 (4)°	*T* = 100 K
*V* = 1354.4 (6) Å^3^	Plate, yellow
*Z* = 4	0.28 × 0.21 × 0.09 mm

### Data collection

**Table:**

Bruker APEX DUO CCD area-detector diffractometer	2647 independent reflections
Radiation source: sealed tube	1663 reflections with *I* > 2σ(*I*)
graphite	*R*_int_ = 0.068
φ and ω scans	θ_max_ = 26.0°, θ_min_ = 2.2°
Absorption correction: multi-scan (*SADABS*; Bruker, 2009)	*h* = −8→10
*T*_min_ = 0.971, *T*_max_ = 0.991	*k* = −10→7
8001 measured reflections	*l* = −23→23

### Refinement

**Table:**

Refinement on *F*^2^	Primary atom site location: structure-invariant direct methods
Least-squares matrix: full	Secondary atom site location: difference Fourier map
*R*[*F*^2^ > 2σ(*F*^2^)] = 0.064	Hydrogen site location: inferred from neighbouring sites
*wR*(*F*^2^) = 0.175	H-atom parameters constrained
*S* = 1.08	*w* = 1/[σ^2^(*F*_o_^2^) + (0.0653*P*)^2^ + 1.5283*P*] where *P* = (*F*_o_^2^ + 2*F*_c_^2^)/3
2647 reflections	(Δ/σ)_max_ = 0.001
193 parameters	Δρ_max_ = 0.33 e Å^−^^3^
0 restraints	Δρ_min_ = −0.40 e Å^−^^3^

### Special details

**Table:**

Experimental. The crystal was placed in the cold stream of an Oxford Cryosystems Cobra open-flow nitrogen cryostat (Cosier & Glazer, 1986) operating at 100.0 (1) K.
Geometry. All esds (except the esd in the dihedral angle between two l.s. planes) are estimated using the full covariance matrix. The cell esds are taken into account individually in the estimation of esds in distances, angles and torsion angles; correlations between esds in cell parameters are only used when they are defined by crystal symmetry. An approximate (isotropic) treatment of cell esds is used for estimating esds involving l.s. planes.
Refinement. Refinement of F^2^ against ALL reflections. The weighted R-factor wR and goodness of fit S are based on F^2^, conventional R-factors R are based on F, with F set to zero for negative F^2^. The threshold expression of F^2^ > 2sigma(F^2^) is used only for calculating R-factors(gt) etc. and is not relevant to the choice of reflections for refinement. R-factors based on F^2^ are statistically about twice as large as those based on F, and R- factors based on ALL data will be even larger.

### Fractional atomic coordinates and isotropic or equivalent isotropic displacement parameters (Å^2^)

**Table:**

	*x*	*y*	*z*	*U*_iso_*/*U*_eq_	
O1	0.8947 (3)	1.0580 (3)	0.56583 (11)	0.0221 (6)	
O2	0.8198 (3)	0.6581 (3)	0.53014 (11)	0.0219 (6)	
O3	0.5846 (3)	1.3849 (3)	0.41496 (11)	0.0234 (6)	
O4	0.1930 (3)	1.2112 (3)	0.22782 (11)	0.0203 (6)	
O5	0.2463 (3)	0.9265 (3)	0.26329 (11)	0.0211 (6)	
C1	0.8927 (5)	0.5329 (4)	0.56403 (17)	0.0249 (9)	
H1A	0.8673	0.4297	0.5539	0.030*	
C2	1.0063 (4)	0.5797 (4)	0.61407 (17)	0.0244 (9)	
H2A	1.0717	0.5167	0.6440	0.029*	
C3	1.0061 (4)	0.7441 (4)	0.61196 (17)	0.0218 (8)	
H3A	1.0719	0.8098	0.6404	0.026*	
C4	0.8920 (4)	0.7884 (4)	0.56070 (15)	0.0177 (7)	
C5	0.8371 (4)	0.9414 (4)	0.53558 (15)	0.0181 (8)	
C6	0.7141 (4)	0.9497 (4)	0.47546 (15)	0.0177 (8)	
H6A	0.6743	0.8590	0.4539	0.021*	
C7	0.6597 (4)	1.0876 (4)	0.45188 (16)	0.0174 (8)	
H7A	0.7039	1.1740	0.4756	0.021*	
C8	0.5399 (4)	1.1191 (4)	0.39374 (15)	0.0157 (7)	
C9	0.5031 (4)	1.2735 (4)	0.37474 (16)	0.0172 (8)	
C10	0.3898 (4)	1.3086 (4)	0.31901 (15)	0.0162 (7)	
H10A	0.3691	1.4115	0.3065	0.019*	
C11	0.3084 (4)	1.1903 (4)	0.28248 (15)	0.0145 (7)	
C12	0.3391 (4)	1.0334 (4)	0.30161 (15)	0.0152 (7)	
C13	0.4544 (4)	1.0014 (4)	0.35535 (15)	0.0155 (7)	
H13A	0.4769	0.8982	0.3668	0.019*	
C14	0.5532 (5)	1.5429 (4)	0.39800 (19)	0.0317 (10)	
H14B	0.6202	1.6080	0.4291	0.048*	
H14C	0.4421	1.5656	0.4036	0.048*	
H14D	0.5759	1.5621	0.3498	0.048*	
C15	0.1539 (4)	1.3692 (4)	0.20725 (16)	0.0190 (8)	
H15C	0.0763	1.3687	0.1670	0.029*	
H15D	0.2495	1.4216	0.1951	0.029*	
H15A	0.1098	1.4221	0.2460	0.029*	
C16	0.2548 (5)	0.7704 (4)	0.28869 (17)	0.0234 (8)	
H16D	0.1717	0.7097	0.2640	0.035*	
H16A	0.2406	0.7694	0.3385	0.035*	
H16B	0.3580	0.7272	0.2806	0.035*	

### Atomic displacement parameters (Å^2^)

**Table:**

	*U*^11^	*U*^22^	*U*^33^	*U*^12^	*U*^13^	*U*^23^
O1	0.0219 (15)	0.0236 (14)	0.0194 (11)	−0.0015 (11)	−0.0062 (10)	−0.0033 (10)
O2	0.0214 (15)	0.0215 (14)	0.0214 (11)	0.0010 (11)	−0.0076 (10)	0.0023 (10)
O3	0.0271 (16)	0.0136 (13)	0.0269 (12)	−0.0014 (11)	−0.0148 (11)	0.0006 (10)
O4	0.0225 (15)	0.0167 (13)	0.0198 (11)	0.0022 (11)	−0.0106 (10)	0.0018 (9)
O5	0.0236 (15)	0.0138 (13)	0.0239 (11)	−0.0038 (11)	−0.0105 (10)	−0.0012 (9)
C1	0.029 (2)	0.0191 (19)	0.0261 (17)	0.0075 (18)	0.0009 (16)	0.0063 (15)
C2	0.020 (2)	0.033 (2)	0.0205 (16)	0.0054 (17)	0.0015 (15)	0.0070 (15)
C3	0.016 (2)	0.030 (2)	0.0187 (15)	−0.0001 (16)	−0.0019 (14)	0.0027 (14)
C4	0.0135 (19)	0.0238 (19)	0.0153 (14)	−0.0026 (16)	−0.0016 (13)	−0.0002 (14)
C5	0.015 (2)	0.024 (2)	0.0155 (15)	0.0011 (16)	−0.0012 (13)	0.0006 (14)
C6	0.014 (2)	0.0200 (19)	0.0183 (15)	−0.0021 (15)	−0.0049 (13)	−0.0002 (13)
C7	0.0129 (19)	0.0218 (19)	0.0174 (15)	−0.0026 (14)	0.0011 (13)	−0.0004 (13)
C8	0.0157 (19)	0.0169 (18)	0.0140 (14)	−0.0004 (15)	−0.0018 (13)	0.0002 (13)
C9	0.0143 (19)	0.0176 (18)	0.0189 (15)	−0.0029 (15)	−0.0032 (14)	−0.0008 (13)
C10	0.0144 (19)	0.0155 (18)	0.0184 (15)	0.0016 (15)	0.0000 (13)	0.0022 (13)
C11	0.0096 (18)	0.0189 (18)	0.0146 (14)	0.0037 (14)	−0.0017 (13)	0.0016 (13)
C12	0.0115 (18)	0.0181 (18)	0.0155 (14)	−0.0002 (15)	−0.0023 (13)	−0.0017 (13)
C13	0.0132 (19)	0.0161 (18)	0.0166 (15)	0.0008 (14)	−0.0035 (13)	0.0006 (12)
C14	0.044 (3)	0.0117 (19)	0.036 (2)	−0.0027 (19)	−0.0158 (18)	0.0021 (16)
C15	0.019 (2)	0.0172 (18)	0.0196 (15)	0.0027 (15)	−0.0046 (14)	0.0063 (13)
C16	0.029 (2)	0.0140 (19)	0.0259 (17)	−0.0021 (16)	−0.0058 (15)	−0.0004 (14)

### Geometric parameters (Å, °)

**Table:**

O1—C5	1.234 (4)	C7—C8	1.453 (5)
O2—C1	1.372 (4)	C7—H7A	0.9300
O2—C4	1.379 (4)	C8—C9	1.405 (5)
O3—C9	1.371 (4)	C8—C13	1.408 (4)
O3—C14	1.418 (4)	C9—C10	1.393 (5)
O4—C11	1.369 (4)	C10—C11	1.379 (5)
O4—C15	1.445 (4)	C10—H10A	0.9300
O5—C12	1.373 (4)	C11—C12	1.416 (5)
O5—C16	1.427 (4)	C12—C13	1.372 (4)
C1—C2	1.347 (5)	C13—H13A	0.9300
C1—H1A	0.9300	C14—H14B	0.9600
C2—C3	1.416 (5)	C14—H14C	0.9600
C2—H2A	0.9300	C14—H14D	0.9600
C3—C4	1.359 (5)	C15—H15C	0.9600
C3—H3A	0.9300	C15—H15D	0.9600
C4—C5	1.462 (5)	C15—H15A	0.9600
C5—C6	1.473 (4)	C16—H16D	0.9600
C6—C7	1.335 (5)	C16—H16A	0.9600
C6—H6A	0.9300	C16—H16B	0.9600
			
C1—O2—C4	106.3 (3)	C11—C10—C9	119.9 (3)
C9—O3—C14	118.1 (3)	C11—C10—H10A	120.1
C11—O4—C15	117.3 (3)	C9—C10—H10A	120.1
C12—O5—C16	116.2 (3)	O4—C11—C10	124.8 (3)
C2—C1—O2	110.8 (3)	O4—C11—C12	114.9 (3)
C2—C1—H1A	124.6	C10—C11—C12	120.2 (3)
O2—C1—H1A	124.6	C13—C12—O5	126.2 (3)
C1—C2—C3	106.3 (3)	C13—C12—C11	118.9 (3)
C1—C2—H2A	126.9	O5—C12—C11	114.9 (3)
C3—C2—H2A	126.9	C12—C13—C8	122.3 (3)
C4—C3—C2	107.4 (3)	C12—C13—H13A	118.8
C4—C3—H3A	126.3	C8—C13—H13A	118.8
C2—C3—H3A	126.3	O3—C14—H14B	109.5
C3—C4—O2	109.2 (3)	O3—C14—H14C	109.5
C3—C4—C5	132.0 (3)	H14B—C14—H14C	109.5
O2—C4—C5	118.8 (3)	O3—C14—H14D	109.5
O1—C5—C4	118.8 (3)	H14B—C14—H14D	109.5
O1—C5—C6	122.7 (3)	H14C—C14—H14D	109.5
C4—C5—C6	118.5 (3)	O4—C15—H15C	109.5
C7—C6—C5	120.0 (3)	O4—C15—H15D	109.5
C7—C6—H6A	120.0	H15C—C15—H15D	109.5
C5—C6—H6A	120.0	O4—C15—H15A	109.5
C6—C7—C8	128.0 (3)	H15C—C15—H15A	109.5
C6—C7—H7A	116.0	H15D—C15—H15A	109.5
C8—C7—H7A	116.0	O5—C16—H16D	109.5
C9—C8—C13	117.2 (3)	O5—C16—H16A	109.5
C9—C8—C7	119.6 (3)	H16D—C16—H16A	109.5
C13—C8—C7	123.1 (3)	O5—C16—H16B	109.5
O3—C9—C10	123.1 (3)	H16D—C16—H16B	109.5
O3—C9—C8	115.5 (3)	H16A—C16—H16B	109.5
C10—C9—C8	121.4 (3)		
			
C4—O2—C1—C2	0.1 (4)	C7—C8—C9—O3	−1.1 (4)
O2—C1—C2—C3	−0.1 (4)	C13—C8—C9—C10	−1.6 (5)
C1—C2—C3—C4	0.1 (4)	C7—C8—C9—C10	179.5 (3)
C2—C3—C4—O2	0.0 (4)	O3—C9—C10—C11	−177.7 (3)
C2—C3—C4—C5	179.1 (3)	C8—C9—C10—C11	1.6 (5)
C1—O2—C4—C3	0.0 (3)	C15—O4—C11—C10	−0.1 (4)
C1—O2—C4—C5	−179.3 (3)	C15—O4—C11—C12	178.5 (3)
C3—C4—C5—O1	−3.5 (5)	C9—C10—C11—O4	178.8 (3)
O2—C4—C5—O1	175.6 (3)	C9—C10—C11—C12	0.3 (4)
C3—C4—C5—C6	177.0 (3)	C16—O5—C12—C13	9.6 (5)
O2—C4—C5—C6	−3.9 (4)	C16—O5—C12—C11	−170.0 (3)
O1—C5—C6—C7	−0.4 (5)	O4—C11—C12—C13	179.2 (3)
C4—C5—C6—C7	179.1 (3)	C10—C11—C12—C13	−2.1 (4)
C5—C6—C7—C8	179.9 (3)	O4—C11—C12—O5	−1.1 (4)
C6—C7—C8—C9	−177.3 (3)	C10—C11—C12—O5	177.6 (3)
C6—C7—C8—C13	4.0 (5)	O5—C12—C13—C8	−177.5 (3)
C14—O3—C9—C10	−1.0 (5)	C11—C12—C13—C8	2.1 (5)
C14—O3—C9—C8	179.6 (3)	C9—C8—C13—C12	−0.2 (5)
C13—C8—C9—O3	177.7 (3)	C7—C8—C13—C12	178.5 (3)

### Hydrogen-bond geometry (Å, °)

**Table:**

*D*—H···*A*	*D*—H	H···*A*	*D*···*A*	*D*—H···*A*
C14—H14B···O2^i^	0.96	2.47	3.363 (5)	154
C15—H15C···O1^ii^	0.96	2.43	3.365 (4)	165

Symmetry codes: (i) *x*, *y*+1, *z*; (ii) *x*−1, −*y*+5/2, *z*−1/2.
